# Characterization of CdTe Films Deposited at Various Bath Temperatures and Concentrations Using Electrophoretic Deposition

**DOI:** 10.3390/ijms13055706

**Published:** 2012-05-11

**Authors:** Mohd Norizam Md Daud, Azmi Zakaria, Atefeh Jafari, Mohd Sabri Mohd Ghazali, Wan Rafizah Wan Abdullah, Zulkarnain Zainal

**Affiliations:** 1 Department of Physics, Faculty of Science, Universiti Putra Malaysia, 43400 UPM Serdang, Selangor, Malaysia; E-Mail: nori_iron85@yahoo.com (M.N.M.D.); mgm.sabri@gmail.com (M.S.M.G.); 2 Advanced Materials and Nanotechnology Laboratory, Institute of Advanced Technology, Universiti Putra Malaysia, 43400 UPM Serdang, Selangor, Malaysia; E-Mails: atefeh.j87@gmail.com (A.J.); wanrafizah@umt.edu.my (W.R.W.A.); 3 Department of Chemistry, Faculty of Science, Universiti Putra Malaysia, 43400 UPM Serdang, Selangor, Malaysia; E-Mail: zulkar@putra.upm.edu.my

**Keywords:** electrophoretic deposition, cadmium telluride, optical properties, numerical expression

## Abstract

CdTe film was deposited using the electrophoretic deposition technique onto an ITO glass at various bath temperatures. Four batch film compositions were used by mixing 1 to 4 wt% concentration of CdTe powder with 10 mL of a solution of methanol and toluene. X-ray Diffraction analysis showed that the films exhibited polycrystalline nature of zinc-blende structure with the (111) orientation as the most prominent peak. From the Atomic Force Microscopy, the thickness and surface roughness of the CdTe film increased with the increase of CdTe concentration. The optical energy band gap of film decreased with the increase of CdTe concentration, and with the increase of isothermal bath temperature. The film thickness increased with respect to the increase of CdTe concentration and bath temperature, and following, the numerical expression for the film thickness with respect to these two variables has been established.

## 1. Introduction

In the past four decades, the phenomenon of electrophoretic deposition (EPD) has been found in traditional ceramic technology [1]. Thin film solar cells using cadmium telluride (CdTe) absorber layers are one of the primary contenders for large-scale commercialization of photovoltaics. Numerous applications based on CdTe have been deployed worldwide such as in radiation detectors, electro-optical modulators and solar cell fabrication [2]. Currently, many techniques are used and have been reported to grow the CdTe thin films such as vacuum evaporation, sputtering, electrodeposition, metal vapor organic deposition, closed spaced sublimation and spray pyrolysis. Among these techniques, EPD has become an attractive and potential technique due to its many advantages: low cost photocell fabrication, easily adapted from the laboratory to industrial scale, no wastage of materials, short deposition time, simple process and higher deposition rate [3]. CdSe nanocrystal films onto dielectric polymer thin film [4] and quantum dot sensitized solar cells with improved efficiency [5] have been prepared by using EPD. The coating can be made in any shape and the film thickness can be controlled by the deposition conditions [6]. A direct electric field is applied to the suspension and, under the appropriate condition the charged powder overcomes inter-particulate repulsions to create a consolidated deposit with high packing density at the electrode of opposite charge [7]. EPD involves the design of a colloidal suspension with powders dispersed with either an electrostatic or electrosteric method [8]. In the previous works on CdTe films, the application of this technique mostly concentrated on one bath temperature and different CdTe powder concentrations [9] or on a defined concentration but with different bath temperatures [10]. The volumetric ratio 1:1 of toluene and methanol has been used to produce a stable colloidal solution [11]. In this study, we characterized CdTe films deposited by the EPD technique varying both the bath temperatures and CdTe concentrations. From the results, the film thickness was numerically expressed with respect to these two variables. This study also evaluates the appropriate working environments for using the EPD technique, the bath temperature and CdTe concentration, for obtaining the appropriate film thickness for any suitable application.

## 2. Experiment

### 2.1. Sample Preparation

Different amounts of CdTe powder (6–7 micron particle size, Alfa Aeser, Ward Hill, MA, USA) were added into separate 10 mL solution mixtures of methanol and toluene. The mixture was ultrasonically agitated for a few minutes to ensure a homogeneous dispersion of CdTe. The volumetric ratio 1:1 of toluene and methanol was used and it was observed that a reasonably stable colloidal solution was produced for quite some duration. The EPD process was carried out at various bath temperatures, from 30 until 60 °C (±0.5 °C), by applying constant 280 V dc voltage over a 5 min deposition time. Depositing for slightly longer than 5 min will produce uneven or bad quality film on the substrate as CdTe colloid will settle down to the bottom of the EPD cell. The two electrodes; ITO on the glass substrate as the working electrode and graphite rod as the counter electrode, were set in parallel over a distance of 1.0 cm to minimize the cell resistance and maximize the current flow [6]. The ITO glasses were preferred to created conductivity, due to the highly transparent surface that reflects infrared rays while allowing visible light and ultraviolet rays to pass through it. The substrate was washed and rinse with methanol and deionized water. After that, it was cleaned using an ultrasonic bath with methanol solution for 10 min and lastly washed with water.

### 2.2. Characterization of Films

The film samples were characterized by UV-Vis spectrometry (Shimadzu-UV 1650PC), and X-ray diffractometry (XRD, PANalytical (Philips), X’Pert Pro PW3040/60). The XRD data were analyzed by X’Pert High Score software for the identification of the crystalline phases in the films. Crystallite size was determined by Scherrer’s equation,

(1) $$D_{p}=\frac{0.94\lambda }{\beta_{1/2}\;\text{cos }\theta }$$

where *β*_1/2_ is the full width at half maximum intensity of the peak in radian, *λ* is the X-ray wavelength and *θ* is the Bragg angle. The film micro-strain, *ɛ*, was calculated from,

(2) $$ɛ=\frac{\beta \;\text{cos }\theta }{4}$$

and the dislocation density, *δ*, defined as the length of dislocation per unit volume of the crystal was evaluated by using,

(3) $$\delta =\frac{1}{D^{2}}$$

Spectral transmittance (*T*) data was recorded using a double beam UV-Vis spectrometer in the wavelength range 200 to 1100 nm. From the spectral data the absorption coefficient, *α*, was calculated by using,

(4) $$\alpha =\frac{2.303}{d}\text{ln}\left(\frac{1}{T}\right)$$

Absorption coefficient can be used to calculate the optical energy band gap (*E_g_*);

(5) $$\alpha =\frac{A{\left(hv-E_{g}\right)}^{n}}{hv}$$

where *A* is constant, *hν* is photon energy; *n* is the value related to either direct or indirect transition and for CdTe is ½ [12]. This band gap energy can be obtained by plotting (*αhv*)^1/2^ *versus hv*. From the plot, the value absorption edge can be obtained and it refers to minimum energy of light being absorbed by the material [13].

The thickness of CdTe films was determined using an atomic force microscope (AFM, Quesant Q-Scope 250) operating in contact mode, with a silicon nitride cantilever. The resulting images are in the form of three dimensional with 20 μm × 20 μm area of the CdTe thin films.

## 3. Results and Discussion

**Figure 1:**
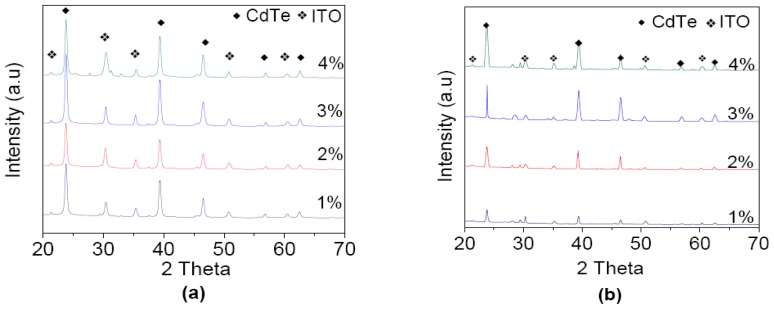
X-ray diffractometer (XRD) patterns of CdTe film at bath temperatures, (**a**) 30 °C; and (**b**) 60 °C from 1 to 4 wt% CdTe concentration.

XRD pattern of CdTe film varying from 1 to 4 wt% concentrations deposited at the different bath temperatures can be seen in Figure 1. It is clear from the pattern that the peak at 2*θ* = 23.8°, 39.4°, 46.7°, 57.4° and 63.5° belong to (111), (220), (311), (410), and (331) plane of cubic CdTe, respectively [10,14]. The results are matched with the JCPDS values (Data file: 75-2086-cubic) for CdTe [15]. Other prominent peaks at the plane (211), (222), (400), (431) and (622) are of the ITO on glass substrate. As the concentration of CdTe increased from 1 to 4 wt%, it can be clearly seen that the peak located at 23.8° (111) was the most prominent. This peak is considerably more intense compared with the rest and often used for highly efficient solar cells [16]. We can see that when the concentration of CdTe increases, the peak intensity also increases and it may be attributed to the increase of the average grain size of the CdTe samples [17].

**Figure 2:**
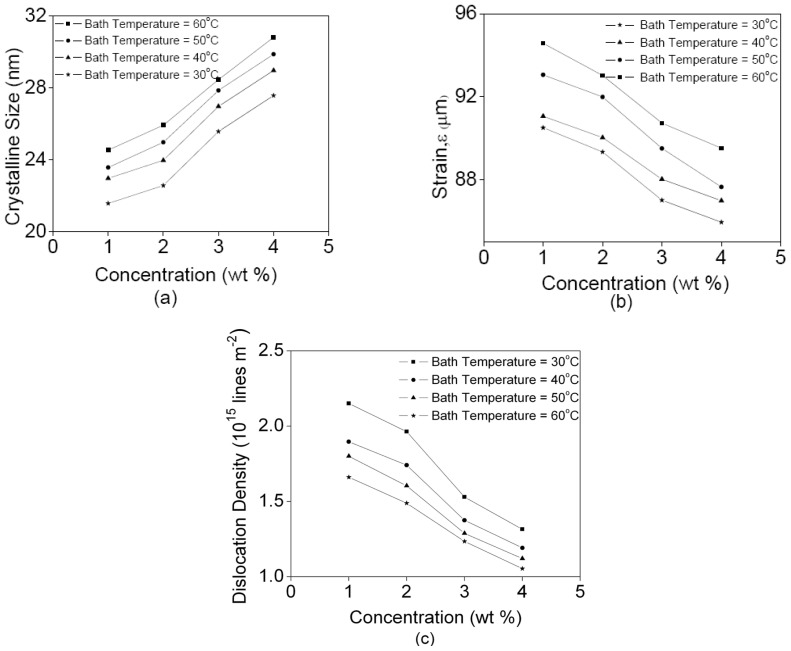
(**a**) Crystallite size; (**b**) strain; and (**c**) dislocation density of CdTe films as a function of CdTe concentration.

Figure 2 shows the crystallite size, micro-strain, and dislocation density at every bath temperature as a function of CdTe concentration. The maximum and minimum crystallite sizes are 30.805 and 21.562 nm for 4 wt% (bath temperature, 60 °C) and 1 wt% (bath temperature, 30 °C), respectively. With the increasing CdTe concentration the crystallite size increases and the micro-strain decreases. The increase in crystallite size at high bath temperature can be interpreted as due to large surface mobility of the depositing atoms. Hence, there is a decrease in the density of nucleation centers and under these circumstances a smaller number of centers start to grow [18]. Dislocation density as well as other lattice defects may also increase the crystallite size. As the crystallite size increases the lattice defect decreases leading to a decrease in micro-strain.

**Figure 3:**
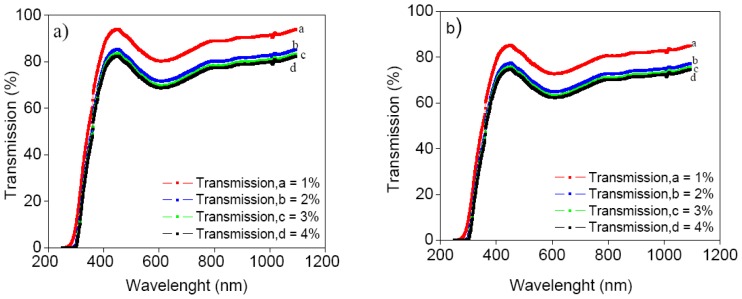
Transmission spectra for bath temperatures (**a**) 30 °C; and (**b**) 60 °C.

The transmittance spectra of the CdTe films coated onto ITO glass substrate for a CdTe concentration from 1 to 4 wt% and bath temperatures of 30 °C and 60 °C are shown in Figure 3. The transmittance peak increases and the window range shifts towards a lower wavelength with the decrease of CdTe concentration. It can be seen that the transmittance has the maxima of 94% and 84% for 1 wt% concentration at bath temperatures of 30 °C and 60 °C, respectively, and decreases with increasing CdTe concentration.

**Figure 4:**
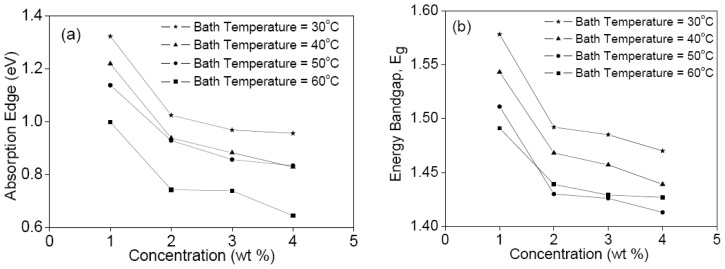
(**a**) Absorption edge and (**b**) optical energy band gap *versus* concentration of a 30 mm × 10 mm area of the CdTe films.

Following from the transmission spectra, we can evaluate the optical energy band gap (*E_g_*) and determine the absorption edge, as can be seen in Figure 4a,b, respectively. Both clearly decrease with the increase of CdTe concentration for each isothermal bath temperature, and also decrease with the increase of isothermal bath temperature, which agrees with previous finding for CdTe thin films [19]. This decrement was attributed to the increase in film thickness and increase in grain size. As the bath temperature increases, the ad-atom mobility also increases, which results in the increase of crystallite size and crystallinity of the films [20]. The same thickness of film will be obtained if the deposition time is constant unless the thickness will increase with the increasing of concentration and bath temperature at a constant of deposition time. However, the increase in bath temperature and concentration can cause a non-uniform film.

**Figure 5:**
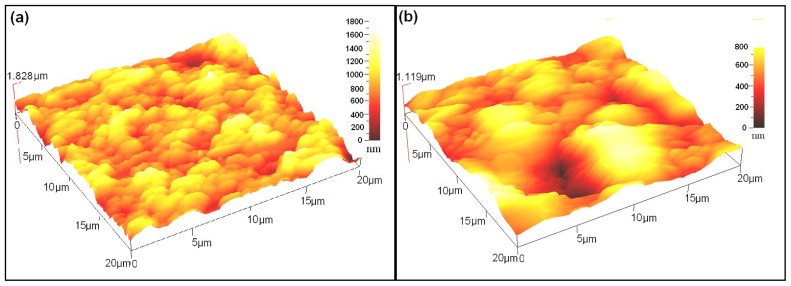
Atomic force microscope (AFM) three dimensional representations of CdTe surface films deposited at bath temperatures (**a**) 30 °C and (**b**) 60 °C for 3 wt% CdTe concentration.

Figure 5a,b display typical AFM micrographs of CdTe films deposited onto ITO glass. The bath temperatures influence the roughness of surface substrate and also the thickness of the films. The films deposited at a bath temperature of 30 °C and at a concentration of 3 wt% have a homogeneously uniform surface and are suitable for solar cell application as their thickness is around 8 μm, Figure 5a. When the bath temperature increases to 60 °C, Figure 5b, it can be seen that the number of grains decreases and grain size increases as a result of increasing of the crystallite size, Figure 2a [21].

## 4. Numerical Relationship of Film Thickness

**Figure 6:**
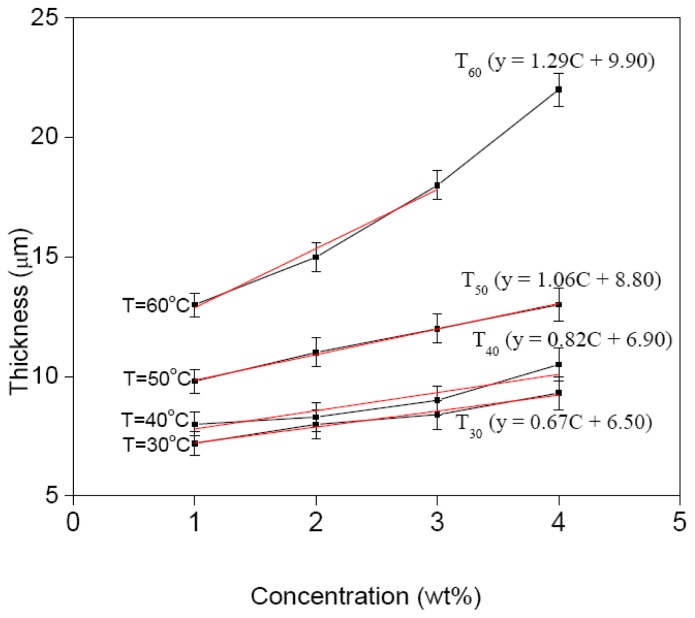
Variation of film thickness with CdTe concentration.

The film thickness increases almost linearly with the increase of CdTe concentration and also increases with the increase of bath temperature, Figure 6. The slight deviation from this linearity is observed at 60 °C and 4 wt%, which is a point that is way off and may be due to sample miss-handling. Based on this figure, we can obtained the numerical relationship for CdTe film thickness *t* with respect to bath temperature *T* and CdTe concentration *c* by assuming it has a linear relationship; *t* = *m*(*T*)*c* + *k*(*T*) in this limited concentration range. Here *m*(*T*) is the gradient and *k*(*T*) is the intercept of each line on the film thickness-axis and both are bath temperature dependent. The *m* and *k* values can be determined by further approximating a linear relationship between film thickness and concentration. Hence, the relationship of *m* and *k* with *T*’s are ln(*m*) = ln(*m*_0_) + *m*_1_*T* and ln(*k*) = ln(*k*_0_) + *k*_1_*T*, respectively. By plotting ln(*m*) and ln(*k*) *versus T* from each line in the plot, the constant values of *m*_0_, *m*_1_ are 0.34253, 0.02222, and *k*_0_, *k*_1_ are 4.20385, 0.01420, respectively. Therefore the numerical expression for the film thickness for CdTe concentrations and bath temperatures in this limited CdTe concentration range can be expressed as

t=0.34253c exp(0.02222T)+4.20385 exp(0.01420T)

This expression is useful to estimate the appropriate environments, *i.e.*, concentration and bath temperature, to produce CdTe film thickness by EPD technique, in the range of 0 to 25 μm. However for comparison, the thickness value obtained by Ebeid *et al*. [22] using EPD technique at only a single temperature of 150 °C and a CdTe concentration of 1.5 wt% is 47 μm, while from the above expression, if extended to this similar temperature, is 49 μm. The estimated thickness differs only slightly by 2 μm or 4%, which is indeed very small, hence this expression may also be applicable for a reasonably high thickness and bath temperature range.

## 5. Conclusions

The CdTe thin film was successfully deposited on an ITO glass substrate by using the EPD technique. The maximum and minimum crystallite sizes are 30.805, 21.562.nm for 4 wt% (bath temperature, 60 °C) and 1 wt% (bath temperature, 30 °C), respectively. The optical energy band gap of film decreases with the increase of CdTe concentrations, and with the increase of isothermal bath temperatures. The film thickness increases with respect to the increase of CdTe concentration and bath temperature. The numerical expression for the film thickness with respect to these two variables has been obtained.
